# Epstein-Barr Virus Induced Gene-2 Upregulation Identifies a Particular Subtype of Chronic Fatigue Syndrome/Myalgic Encephalomyelitis

**DOI:** 10.3389/fped.2019.00059

**Published:** 2019-03-13

**Authors:** Jonathan R. Kerr

**Affiliations:** Department of Microbiology, West Suffolk Hospital Foundation Trust, Bury St Edmunds, United Kingdom

**Keywords:** Chronic Fatigue Syndrome, Myalgic Encephalomyelitis, Epstein-Barr virus, Epstein-Barr virus induced gene 2, autoimmune, microarray, real-time polymerase chain reaction

## Abstract

Chronic Fatigue Syndrome/Myalgic Encephalomyelitis (CFS/ME) is a chronic multisystem disease characterized by a variety of symptoms, and exhibits various features of an autoimmune-like disease. Subtypes are well recognized but to date are difficult to identify objectively. The disease may be triggered by infection with a variety of micro-organisms, including Epstein-Barr virus (EBV). A subset of CFS/ME patients exhibit up regulation of EBV virus induced gene 2 (*EBI2*) mRNA in peripheral blood mononuclear cells (PBMC), and these patients appear to have a more severe disease phenotype and lower levels of EBNA1 IgG. *EBI2* is induced by EBV infection and has been found to be upregulated in a variety of autoimmune diseases. *EBI2* is a critical gene in immunity and central nervous system function; it is a negative regulator of the innate immune response in monocytes. Its heterogeneous expression in CFS/ME could explain the variable occurrence of a variety of immune and neurological abnormalities which are encountered in patients with CFS/ME. The *EBI2* subtype occurred in 38–55% CFS/ME patients in our studies. Further work is required to confirm the role of EBV and of *EBI2* and its oxysterol ligands in CFS/ME, and to identify the most practical means to identify patients of the EBI subtype. There are two *EBI2* antagonists currently in development, and these may hold promise in the treatment of CFS/ME patients of the EBI subtype.

## Introduction

Chronic Fatigue Syndrome/Myalgic Encephalomyelitis (CFS/ME) is a chronic multisystem disease characterized by at least 6 months of fatigue and a variety of other symptoms, including headache, sore throat, muscle pain, joint pain, muscle weakness, post-exertional malaise, sleep abnormalities, and secondary anxiety and depression (1). It is most likely that heterogeneity in CFS/ME is the reason that although there have been many immune and other abnormalities found in patients, none are universal and so there are currently no biomarkers of CFS/ME *per se*. This is used as evidence against a biological pathogenesis of the disease, however, the most plausible explanation is that CFS/ME is a heterogeneous autoimmune-like disease with a variety of subtypes, a phenomenon typical of autoimmune disease.

Particular problems are, first, that the CFS/ME is unique as a chronic autoimmune-like disease, in that there is no objective means to confirm the diagnosis. Secondly, there are a variety of names and diagnostic criteria some of which do not exclude major depression, as is required by the CDC criteria (1) and Canadian criteria (2). Thirdly, there has been a push to combine biological and unrelated psychological approaches in CFS/ME research. Of course, psychological aspects must be included, but only in terms of what we know is relevant to current knowledge of CFS/ME. Namely, that psychological stress can trigger the disease, and that anxiety and depression are secondary phenomena in CFS/ME (1, 2).

By analogy to autoimmune diseases, psychological aspects are known to be almost universal, for example, anxiety in ulcerative colitis, rheumatoid arthritis, psoriasis, asthma, etc. And the anxiety is believed to underlie relapses and flare-ups which may precipitate hospital admission and use of immune-modifying treatments in a variety of autoimmune diseases.

In this paper, I will review the heterogeneity of CFS/ME, its clinical presentation and triggering factors including Epstein-Barr Virus (EBV) reactivation, CFS/ME as an autoimmune-like disease, Epstein-Barr virus induced (EBI) gene 2 (*EBI2*) dysregulation in CFS/ME, the “EBI subtype” and the future possibility for therapeutic pharmacological EBI2 antagonism in CFS/ME patients.

## Heterogeneity of CFS/ME

Heterogeneity among CFS/ME patients is well recognized. There are clear differences in type of onset (sudden vs. gradual), duration of illness, and different types of illness (predominant pain, predominant flu-like illness, predominant neurological type illness, etc.). In a comparative review of systemic and neurological symptoms in 12 outbreaks of CFS/ME, epidemic neuromyasthenia and ME, marked heterogeneity in the range of neurological symptoms was observed (3), and outbreaks could be grouped into four levels of increasing neurological involvement. Janal et al. (4) subtyped CFS/ME patients according to “minor” symptoms, and identified three subtypes; neurological, musculoskeletal, and infectious. Extreme scores in one or more of these factors accounted for 66% of the sample. Depression and anxiety were not more prevalent in any particular subtype. Jason et al. (5) analyzed data from 18,675 CFS/ME patients and found strong evidence for the existence of a variety of subtypes based on sociodemographic status and disability. Jason et al. (6) have outlined the importance of subtyping of CFS/ME patients, both for the study of pathogenesis and for response to available treatments. These authors identified CFS/ME subtypes based on level of disability, viral, immune, neuroendocrine, neurological, autonomic, and genetic aspects. Proper identification and study of CFS/ME subtypes has been hampered by the lack of concensus as to how to diagnose the disease.

## CFS/ME as a Chronic flu-Like Illness Triggered by Virus Infection

At its most simple, CFS/ME can be considered to be a chronic flu-like illness triggered by virus infection, and that the symptoms of CFS/ME are those of a resulting flu-like illness (fatigue, impairment in short term memory or concentration, sore throat, muscle pain, joint pain without swelling or redness, headaches, unrefreshing sleep, and post-exertional malaise) (7). Psychological symptoms (anxiety and depression) are common during flu-like illnesses, and are secondary in otherwise healthy persons. There is a biological basis for such secondary psychological symptoms in that circulating proinflammatory cytokines result in activation of glial cells in the brain and secretion of proinflammatory cytokines by these cells, causing the symptoms of depression, lethargy and anxiety (8). We are all familiar with such short term illness and its symptoms, including the secondary psychological symptoms. We are familiar with the sickness behavior we exhibit during these short term illnesses. For example, we prefer to go to bed early rather than stay out late (8). The principal difference between a short term flu-like illness secondary to virus infection, and CFS/ME, is the duration, severity and the global effect on the lives of patients. In the case of CFS/ME, generally 6 months of symptoms are required (1), while a short term flu-like illness would typically last < 2 weeks.

## Psychological Stress is Key to Virus Transmission, Infection, and CFS/ME

For any given virus infection, outcomes vary according to many factors. However, for the purposes of CFS/ME, the key factor is psychological stress. It has been shown for a variety of viruses that psychological stress is necessary for successful virus transmission from one person to another (9). Furthermore, it has been shown that psychological stress is necessary for symptoms to develop after successful transmission, as opposed to asymptomatic infection (9). It is well known that psychological stress is key in the reactivation of herpes viruses, and this precedes the recurrence of cold sores (herpes simplex virus) (10), shingles (varicella-zoster virus) (11), and Epstein-Barr virus (EBV) (12, 13). Psychological stress has been shown to be important in triggering a large proportion of cases of CFS/ME, and this fits perfectly with a viral pathogenesis. Psychological stress is universal and is expected under various circumstances, for example, student examinations, loss of a parent or partner, etc.

## Important Microbial Triggers of CFS/ME

Micro-organisms which have been documented to trigger CFS/ME include EBV, enteroviruses, cytomegalovirus (CMV), human herpesvirus-6 (HHV-6), human parvovirus B19, hepatitis C, *Chlamydia pneumonia*, and *Coxiella burnetii* (Table 1). EBV is known to infect 90% humans, the majority of which become infected in childhood due to transmission in oral secretions. Following acute infection, EBV persists life-long. It is not clear what percentage of CFS/ME patients are infected with EBV, but in one UK study, it was 90% (34). It is recognized that for each microbial trigger of CFS/ME, that there are a variety of possible clinical outcomes of acute infection, including CFS/ME. It is also recognized, that of those patients who suffered an acute microbial infection which led to development of CFS/ME, there are a number of possible resulting CFS/ME phenotypes, and that this varies depending on other, as yet unknown factors. Therefore, of those patients who developed CFS/ME following parvovirus B19 infection, for example, some will have a CFS/ME phenotype with predominant musculoskeletal pain, while others will have less predominant pain, and more problems with sleep, memory, and concentration, for example. Therefore, there is a lack of correlation between the particular microbial or other trigger and the resulting CFS/ME phenotype.

**Table 1:** Microbial infections which have been shown to trigger CFS/ME.

**Micro-organism (virus or bacterium)**	Microbial persistence after the acute phase*	**Treatment**	**References**
Enteroviruses	No	Interferons α, γ	(14–19)
Epstein-Barr virus (EBV)	Yes	Valacyclovir, Valgancyclovir	(20–25)
Cytomegalovirus (CMV)	Yes	Cidofovir, Human normal immunoglobulin (IVIG)	(18)
Human herpes virus-6	Yes	Cidofovir	(18, 26, 27)
Parvovirus B19	Yes	IVIG	(28–31)
Hepatitis C	Yes	Interferon / ribavirin	(18)
*Chlamydia pneumoniae*	No	Tetracycline, clarithromycin	(18, 32)
*Coxiella burnetii*	Yes	Tetracyclines	(33)

* *The majority of those micro-organisms important in triggering CFS/ME have been shown to persist following the acute phase*.

## CFS/ME Exhibits Features of an Autoimmune Disease

A variety of features suggest that CFS/ME may be an autoimmune-like disease. CFS/ME may be triggered by virus infection, and its course characterized by a typical “viral” flu-like illness (7). These observations have led to the recognition that the immune response plays a large and significant role in the pathogenesis of the disease. There are striking similarities between CFS/ME and various autoimmune diseases, for example, Multiple Sclerosis (MS). And, the existence of subtypes of CFS/ME is a further parallel to autoimmune diseases, in which subtypes are well recognized. There are various examples of subtypes of autoimmune diseases exhibiting specific pathogenetic mechanisms, such that particular subtypes of particular autoimmune diseases may be amenable to specific treatments while other subtypes of the same autoimmune disease are not. Studies have demonstrated a variety of immune abnormalities in CFS/ME patients (Table 2), many of which are also found in patients with autoimmune disease. A variety of autoantibodies have been demonstrated in serum of CFS/ME patients including those against nuclear and membrane structures, neurotransmitters and their receptors, cytoplasmic intermediate filaments, EBV dUTPase, and neoepitopes resulting from oxidative or nitrosative damage (53). There is considerable co-morbidity of CFS/ME with other immune or autoimmune diseases, including fibromyalgia (30–77%), postural orthostatic tachycardia syndrome (POTS) (11–40%), Hashimoto's thyroiditis (17–20%), and a family history of an autoimmune disease (18–41%) (53). We have also found upregulated EBI2 mRNA expression in a subset of CFS/ME patients which also occurs in autoimmune diseases (see below).

**Table 2:** Various immunological abnormalities which have been reported in CFS/ME patients.

**Positive study findings in CFS**	**Negative study findings**	**References**	
		**Positive findings**	**Negative findings**
Significant increase in B cells expressing CD20 and CD21	No difference in B cells between CFS and Normals	(35, 36)	(37, 38)
An increase in CD8+/HLADR+ and CD8+/CD38+ T cells	No difference in CD8+/HLADR+ and CD8+/CD38+ T cells	(35, 36)	(38)
Increased T cell differentiation	No increased T cell differentiation	(39)	(38)
NK cell dysfunction		(40)	
Reduction in CD3-/CD16+ and CD57+/CD56+ NK cells with an expansion of the CD8+/CD56+ and CD16-/CD56+ NK subsets and total circulating B cells		(36)	
Deficiency in NKH.1+ T3 cell numbers and decreased NK cell function in patients with CFS who had evidence of EBV reactivation		(41)	
Deficiency in cellular immunity with reduced cytotoxicity of NK cells with increased NK numbers		(35)	
Total NK numbers normal, with decreased NK cell activity as compared to normal (CFS family)		(42, 43)	
Decreased antibody-mediated cellular cytotoxicity (ADCC)		(43)	
Th2 profile of CD4 helper T cell responsiveness		(44–46)	
Reduced TGF1 mRNA expression		(47)	
Increased neutrophil apoptosis		(48)	
Deficiency of IgG1 in 2 CFS patients		(49)	
Deficiency of IgG1 and IgG3 in CFS compared with healthy sedentary controls. IgG1 and IgG3 were even lower in CFS with concurrent axis-I depression as compared with CFS itself		(50)	
Deficiency of IgG1, IgG3, and IgG4		(51)	
Deficiency of IgG3		(52)	

Two clinical trials of monoclonal anti-CD20 antibody, rituximab, in CFS/ME patients demonstrate partial or complete benefit in 60%, and in some of these the remission was sustained. This treatment, in both trials, exhibited a delayed onset of response of ~4 months, suggesting that benefit was not directly mediated by CD20 depletion, but by plasma cell depletion followed by washout of short-lived autoantibodies (54, 55).

## EBV Reactivation is a Model for Psychological Stress and Triggering of CFS/ME

Acute EBV infection has been shown to be an important virus trigger of CFS/ME (20–22). Study of acute EBV infection in medical students shows that EBV preferentially reactivates during the psychologically stressful time of examinations as compared with other less stressful periods of the academic year (56). This has also been shown for military recruits in training at examination times (57). This reactivation is also paralleled by changes in a large variety of immune markers of cellular immunity which are important in the long-term control and suppression of replication of persistent and asymptomatic EBV in the normal person (12, 13). These studies elegantly document the importance of the balance between persistent EBV and cellular immune system competency which can be disrupted by psychological stress, leading to reactivation and replication of EBV, and subsequent manifestation of symptoms of EBV infection, which if the stress is maintained, can become prolonged and lead to CFS/ME and other diseases, such as nasopharyngeal cancer and post-transplant lymphoproliferative disorder (PTLD) (58). The various types of stress that can adversely affect the efficacy of the cellular immune response include marital stress (59), student examination stress (56), attachment anxiety or fear of abandonment and rejection (60), loneliness (61), etc. But physical stress by itself does not have this effect (62).

Psychological stress triggers release of glucocorticoids which activate EBV lytic infection through the upregulation of the immediate early BZLF1 gene expression (63). The cause of the pro-inflammatory state with EBV reactivation is the EBV-encoded deoxyuridine triphosphate nucleotidohydrolase (dUTPase) which modulates innate immunity in human primary monocyte-derived macrophages through toll-like receptor (TLR)-2 signaling leading to NF-κB activation and the production of pro-inflammatory cytokines. EBV dUTPase induces sickness responses in mice (64). Restraint stress (unavoidable stress which causes autonomic and behavioral changes) results in impairment of learning and memory which is due to expression of EBV dUTPase (65).

CFS/ME patients exhibit prolonged raised antibody titers against EBV dUTPase and EBV DNApol which are neutralizing, and may be used to identify CFS patients in which their disease pathogenesis is due to ongoing EBV reactivation (66). However, global screening of serum antibody responses to an EBV peptide array in serum of CFS/ME patients compared with controls revealed strikingly similar patterns (67).

## Epstein-Barr virus (EBV) Induced GENE 2 (*EBI2*)

Epstein-Barr Virus (EBV) induced gene 2 (*EBI2*) is a G-Protein Coupled Receptor (GPCR), also known as GPR183, which was originally identified as the main induced gene in Burkitt's Lymphoma cells upon infection with EBV (68). *EBI2* has been found to be highly expressed in peripheral blood mononuclear cells (PBMC) (B, T, NK, monocytes, and granulocytes) during EBV reactivation (68–70), is a regulator of B cell partitioning in tissues of the lymphoid system and is critical for T-cell mediated antibody responses (71–74) and inflammation (71, 72, 75). *EBI2* has also been found in dendritic cells and monocytes (76). *EBI2* is activated by oxysterols and pertussis toxin-sensitive heterotrimeric G proteins, resulting in decreased cyclic AMP, mobilization of calcium and activation of the extracellular signal related kinase (ERK) pathway (69, 77). High affinity *EBI2* agonists are the oxysterol, 7α 25-dihydroxycholesterol (7α25HC) and related compounds (78, 79). Activation of *EBI2* with 7α25HC results in a wide range of functional responses including cell migration and calcium mobilization (78, 79). 7α25HC is synthesized from cholesterol (80). Other oxysterols also activate EBI2 but with lower potency (78).

*EBI2* also plays an important role in the central nervous system (69). Astrocytes are the macrophages of the brain and protect it against invading pathogens and astrocyte abnormalities are implicated in multiple sclerosis (MS), Parkinsons Disease (PD), Amyotrophic Lateral Sclerosis (ALS), and Alzheimer Disease (AD). Oligodendrocytes are the main cells involved in myelination of nerve fibers of the brain and are implicated in leukodystrophies and leukoencephalopathies. Cholesterol is a crucial component of myelin and cholesterol deficiency results in motor symptoms (81). Abnormal levels of oxysterols have been found in AD, MS and Experimental Allergic Encephalomyelitis (EAE). Mutations in CYP7B1 gene have been demonstrated in Spastic Paraplegia Gene 5 (SPG5) and lead to lesions of upper motor neurones, periventricular areas and subcortical white matter (82, 83). Brain cholesterol is synthesized in the brain as it can't traverse the blood brain barrier (BBB) (84, 85). The cholesterol metabolite, 24(S)-hydroxycholesterol (24(S)HC) is a brain-specific oxysterol which is thought to be synthesized exclusively in the brain and secreted into the circulation via the BBB to maintain steady levels of brain cholesterol (85). Circulating 24(S)HC is believed to be a biomarker for brain cholesterol homeostasis and neurodegenerative disease (MS, PD, AD) (85–87). Oxysterols also have detrimental effects on myelin and oligodendrocyte viability (88, 89).

Dysregulation of *EBI2* expression has been demonstrated in EBV infection (68, 69, 73, 74, 76), melanoma metastasis, lymphoblastic leukemia, glioblastoma, bone cancer metastasis, systemic lupus erythematous, chronic rhino sinusitis with nasal polyps, Type 1 Diabetes (69), and CFS/ME (90) (see below). Aberrant oxysterol signaling has been demonstrated in Multiple Sclerosis, Experimental Allergic Encephalomyelitis (EAE), Alzheimer's disease, Parkinsons Disease, Motor Neurone Disease, Cerebrotendinous Xanthomatosis, Hereditary spastic paraplegia type 5 (SPG5), Huntingdon Disease, Age related macular degeneration, atherosclerosis, Inflammatory bowel disease, and osteoporosis (69). *EBI2* regulates several genes, important in monocyte function, which are important in the pathogenesis of glioblastoma multiforme and Type 1 Diabetes Mellitus. Knock down of the *EBI2* gene in rat monocytes, results in upregulated IRF7 expression. As IRF7 is a critical regulator of the type 1 interferon response, this suggests that *EBI2* is a negative regulator of the innate immune response in macrophages (91). However, the precise role played by *EBI2* in EBV infection remains to be clarified (68, 69).

## Expression of EBI2 in CFS/ME

We have previously found that CFS/ME patients exhibit significantly upregulated expression of *EBI2* in PBMC as compared with normal controls, in gene expression arrays and reverse-transcriptase polymerase chain reaction (RT-PCR) confirmation assays (90). *EBI2* mRNA (NM_004951) expression was found to be significantly upregulated by a factor of 1.3 in microarray experiments using PBMC from 25 CFS patients vs. 50 normal human controls matched 2:1. This upregulation was confirmed using RT-PCR in 55 CFS/ME patients vs. 75 normal human controls at a fold difference of 3.44 (*P* = 0.0012) using ABI assay number Hs00270639_s1 (90). In this study, *EBI2* was found to be unregulated in 55% CFS/ME patients, one of whom was a 26 year old woman whose CFS/ME had been triggered by laboratory documented EBV infection 10 years prior (90). All those with raised expression of EBI2 were also positive for serum anti-VCA IgG.

**Figure 1:**
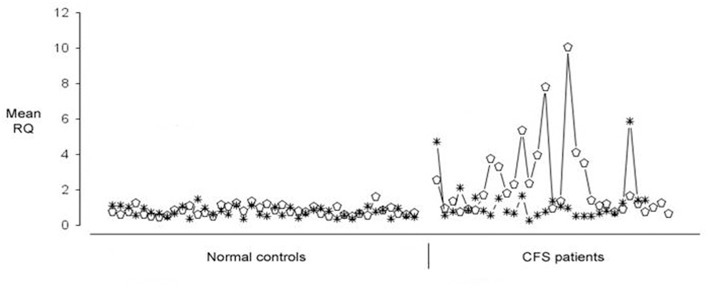
Expression of Epstein-Barr Virus (EBV) induced gene 2 (*EBI2*) (open ellipse) and Neuropathy Target Esterase (NTE) (asterisk) genes in 40 healthy blood donors (shown on the **Left**) and 31 Chronic Fatigue Syndrome/Myalgic Encephalomyelitis (CFS/ME) patients (shown on the **Right**). Upregulated *EBI2* mRNA expression was demonstrated in 12 of 31 CFS/ME patients, and in none of the controls.

Microarray experiments identified 88 human genes which were upregulated in CFS/ME, and the RT-PCR expression data on these 88 human genes in 52 CFS patients was then clustered and this identified seven gene expression subtypes. These gene expression derived subtypes differed significantly in measures of clinical symptomatology and neurocognitive functioning (90). Of these 88 human genes which were differentially expressed in CFS patients, they could be divided into two groups each with 44 genes, one group of which showed quite predictable up regulation across most CFS/ME patients and in the other group, the expression was much more variable. *EBI2* was one of those which was more variably upregulated in PBMC of CFS/ME patients. This is illustrated in Figure 1, in which 12 of 31 (38%) patients exhibited *EBI2* up regulation as compared with none of 40 normal controls.

Although it was known in 2008 that *EBI2* was upregulated in PBMC of CFS/ME patients (90), the significance was not understood at the time as little was known about the gene and its function. However, with the recent identification of *EBI2* as a critical regulator of the immune response with importance for a variety of autoimmune diseases and cancer, its significance in the pathogenesis of CFS/ME has been recognized.

## Possible Link Between *EBI2* Expression, EBV Latency, and Clinical Severity in CFS/ME

As part of a separate study of microbial infections in CFS/ME, we analyzed EBV antibody markers in 117 CFS/ME patients which had been grouped into eight subtypes (A-H) based on clustering of RT-PCR expression data for 88 CFS/ME-associated genes (34). These 117 CFS/ME patients included 55 CFS/ME patients who had been included in the previous study (90), 56 CFS/ME patients who had not previously been studied and six whose disease had been triggered by acute Q fever (QFS) (34). The CFS/ME patients exhibited 90% EBV seropositivity which is to be expected (34).

**Figure 2:**
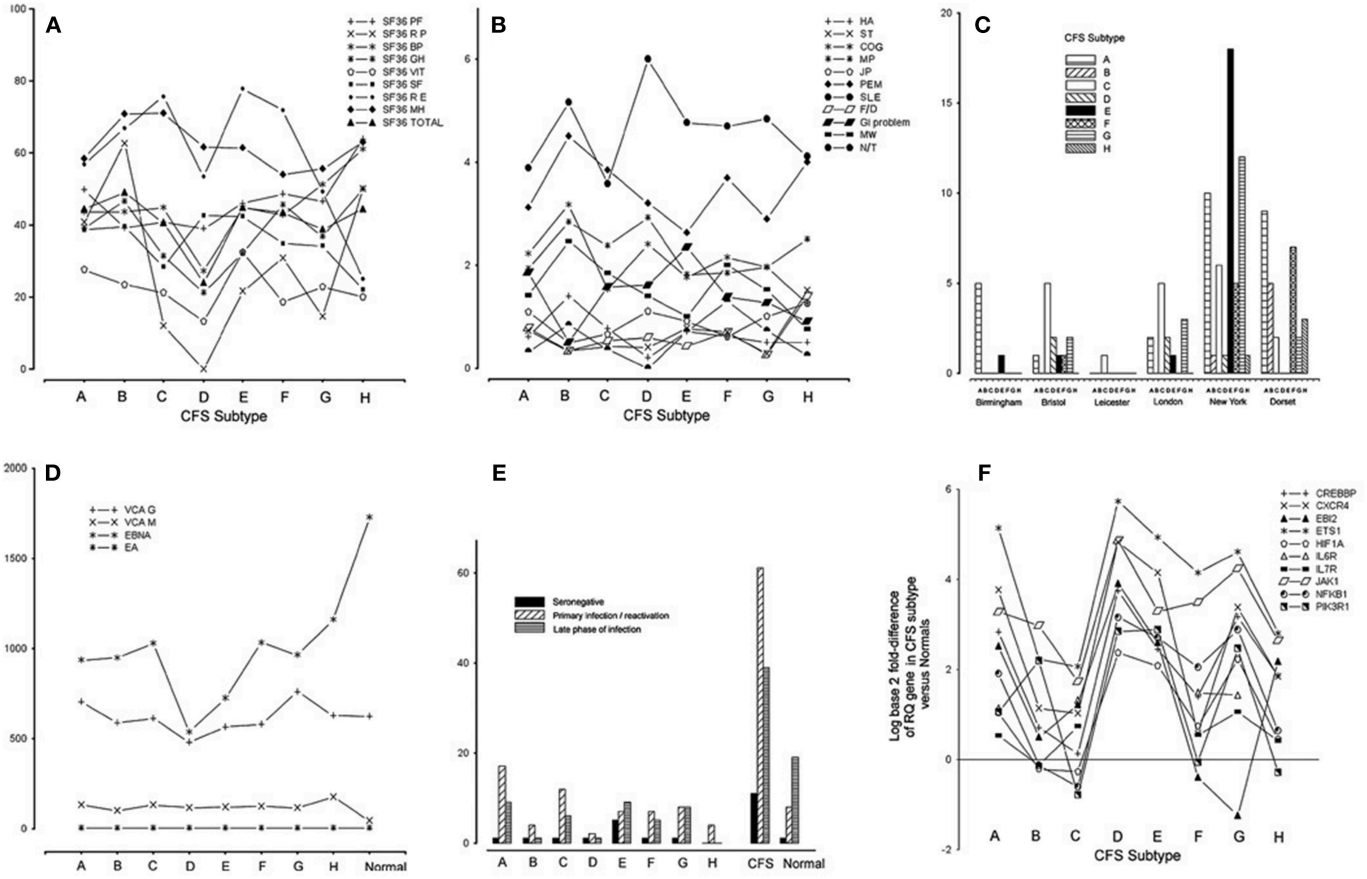
**(A)** Medical Outcomes Survey Short Form-36 (SF36) domain and total scores for each chronic fatigue syndrome/myalgic encephalomyelitis (CFS/ME) subtype: physical function (PF), physical role (RP), bodily pain (BP), general health (GH), vitality (VIT), social functioning (SF), emotional role (RE), mental health (MH), and total score (Total). **(B)** Scores indicating occurrence and severity of 11 clinical symptoms for each CFS/ME subtype: headache (HA), sore throat (ST), swollen glands (GLA), cognitive defect (COG), muscle pain (MP), joint pain (JP), muscle weakness (MW), post-exertional malaise (PEM), sleep problems (SLE), fainting/dizziness (F/D), gastrointestinal complaints (GI), numbness/tingling (N/T), spatial span (SSP), verbal recognition memory (VRM). **(C)** Histogram showing the numbers of CFS/ME patients of each subtype occurring in each of the six geographical locations. **(D)** Epstein-Barr virus (EBV) antibody titers [viral capsid antigen (VCA) IgM, VCA IgG, early antigen (EA) IgG, Epstein-Barr nuclear antigen (EBNA) IgG] in each CFS/ME subtype and the normal comparison group. **(E)** Distribution of categories of EBV serostatus (seronegative, primary/re-activation, late phase of infection) in the CFS/ME subtypes, A-H, in CFS/ME (all subtypes combined) and in normal controls. **(F)** Log (base 2) of fold-difference values of 10 human genes known to be important in EBV infection, in eight CFS subtypes (A-H). Reproduced from Figure 1 of reference no. 34 with permission from BMJ Publishing Group Ltd. (License number 4413611406714).

Subtype D was the most interesting in terms of EBV infection markers and clinical phenotype (34). Subtype D consisted only of females and had the most severe clinical phenotype, with the lowest functional level on SF-36 scoring (physical role, vitality, general health, bodily pain, and total score) and a high frequency of muscle pain and sleep problems (Figures 2A,B). *EBI2* was expressed at the highest level in PBMC of subtype D patients [fold difference (CFSME/Normal), 14.93] as compared with the other subtypes [Mean fold difference (CFSME/Normal), 3.004] (34). There were no discernible differences in EBV antibody markers [viral capsid antigen (VCA) IgM and IgG, early antigen (EA) IgG, and Epstein-Barr nuclear antigen (EBNA) IgG] between subtypes, except that in Subtype D, CFS/ME patients had a markedly reduced titer of EBNA IgG (Figure 2D).

Epstein-Barr virus nuclear antigen 1 gene (*EBNA1*) is important in establishing and maintaining the altered state that cells undergo during EBV infection, and is the only EBV protein found in all EBV-associated malignancies. *EBNA1* has a glycine-alanine repeat which stabilizes the protein, prevents its breakdown, impairs antigen processing, and MHC class I-restricted antigen presentation, resulting in inhibition of the CD8-restricted cytotoxic T lymphocyte response against EBV infected cells, thus favoring latency (92). The finding that CFS/ME patients of subtype D with *EBI2* mRNA upregulation had lower titers of EBNA IgG than the other subtypes (Figure 2D), supports the idea that subtype D is associated with a higher prevalence of EBV latency, as lytic infection is required to expose this antigen to circulating lymphocytes, a necessary step in developing serum EBNA IgG positivity.

In one study, it was shown that in 10% CFS/ME patients, EBNA IgG titers were low or absent (93). Multicolor flow cytometry revealed that the frequencies of EBNA-1-specific triple TNF-α/IFN-γ/IL-2 producing CD4(+) and CD8(+) T-cell subsets were significantly diminished in CFS/ME patients (93).

Within the CFS/ME-associated gene signature of 88 human genes, 12 had recognized associations with EBV infection. One of these was *EBI2*, as discussed, and the others were *NFKB1, EGR1, ETS1, GABPA, CREBBP, CXCR4, HIF1A, JAK1, IL6R, IL7R*, and *PIK3R1*. Striking associations were found for these 12 genes across subtypes, and subtype D had the highest levels of all of them in PBMC (Figure 2F).

## Heterogeneous *EBI2* Upregulation May Contribute to the Variability of Immune and Neurological Abnormalities in CFS/ME

The following have been variably been found in CFS/ME patients; increase in the number of circulating B lymphocytes, increase in activated T lymphocytes, reduction in NK cell numbers and/or function, deficiency in antibody-mediated cellular cytotoxicity (ADCC), Th2 profile of helper T cell responsiveness, reduced TGF1 expression, increased neutrophil apoptosis, and deficiencies in particular IgG subsets (Table 2). During the normal humoral immune response, activated B cells upregulate *EBI2* which mediates their journey to the outer follicle where they interact with T helper cells. After CD40 engagement, *EBI2* expression results in cells moving away from the B-T boundary toward the outer and inner areas of the follicle (73), differentiation into plasmablasts, and mounting of a rapid antibody response. Some B cells move to the central follicle, differentiate into germinal center B cells, to later exhibit antibody affinity maturation (71–75). A higher than normal expression of *EBI2* could result in both increased numbers of B cells in the circulation, and in reduced T cell help and therefore deficiencies in particular IgG subsets, and reduced antibody affinity maturation. EBV reactivation is associated with expansion of differentiated and activated CD4 and CD8 T lymphocytes and later with decline in these cells as exhaustion takes over (94), and so the timing of EBV infection in CFS/ME will affect research studies on immune abnormalities. NK cells are important in defense during the early stages of primary EBV infection (94). Innate immune control of lytic EBV infection by early differentiated NK cells was found to attenuate infectious mononucleosis (IM) (95). It has been proposed that NK cells are important in the long-term control of EBV (96), which may account for the variable findings related to NK numbers and function in CFS/ME, as not all CFS/ME patients will have EBV reactivation at the time of sampling.

Abnormalities in white matter, gray matter and in cerebral perfusion have been found in CFS/ME patients, and these occur in a similar presentation to those of MS patients. Both CFS/ME and MS patients have reduced cerebral perfusion, gray matter reduction and white matter hyper intensities, although individual patients are variably affected (97). As *EBI2* expression and oxysterol dysregulation have been linked with the pathogenesis of MS (69), and as MS is characterized by a relapsing and remitting course, in which subtypes exist and in which *EBI2* is variably upregulated in some of these, it is logical to suggest that heterogeneous *EBI2* expression may similarly play a role in the neurological abnormalities found in CFS/ME.

Although we know that there are a variety of immune abnormalities which occur with regularity in CFS/ME patients, these do not occur invariably, and none can be used as a marker for the presence of the disease. However, the upregulation of *EBI2* in a subset of CFS/ME patients may contribute to this phenomenon.

## EBI2 Modulators

*EBI2* is a key receptor in B, T and dendritic cells, modulating the T and B cell response to blood borne antigens (76). As *EBI2* and/or its oxysterol ligands are upregulated in B cell malignancies and autoimmune diseases (Type 1 Diabetes, rheumatoid arthritis, systemic lupus erythematosus, and multiple sclerosis), two *EBI2* modulators are being developed. GSK682753A is a small molecule, potent EBI2 antagonist which blocks 7a25HC stimulation of the *EBI2* receptor in a recombinant system (98). NIBR189 is a potent selective antagonist of *EBI2*, which has been developed in paradigms relevant to cardiovascular disease (99).

## Limitations of the Hypothesis that EBI2 Upregulation is Important in a Subset of CFS/me Patients

The raw data underpinning the present review were generated by only one research group. Therefore, it would be important that these findings are replicated in additional CFS/ME patients and normal controls by independent research groups.

Elevated levels of antibodies to EBV VCA, EA, and DNase have been reported to occur in CFS/ME patients (20, 21, 66, 100–105) albeit inconsistently (106–108). However, it is important to understand that EBV antibody markers may associate with CFS/ME, but this does not prove that EBV has triggered the disease in those particular cases. The seroprevalence of EBV in the general population and in CFS/ME patients is ~90%. And the proportion of CFS/ME patients with EBI2 upregulation was found to be between 38 and 55% CFS/ME patients, all of whom had IgG to EBV VCA. For a disease in which a variety of microbial triggers are recognized, our findings are consistent with the hypothesis that upregulation of EBI2 is important in the pathogenesis of disease in a subset of CFS/ME patients. However, this hypothesis remains wholly unproven and it is not understood what factors, in addition to EBV infection, are required for upregulation of EBI2 in an individual patient.

Although EBI2 was found to be the most upregulated gene in EBV-infected Burkitt lymphoma cells (68) and has been shown to be important in a variety of autoimmune diseases and cancers, the particular role of EBI2 in the pathogenesis of EBV infection still remains to be elucidated. Therefore, the possible pathogenetic role of EBI2 upregulation in CFS/ME patients remains speculative at present.

## Conclusion

CFS/ME is a heterogeneous disease which is frequently triggered by virus infection, including EBV. Subtypes are well recognized but to date are difficult to identify objectively. Evidence is presented to document that a subset of CFS/ME patients exhibit up regulation of *EBI2* mRNA in PBMC. *EBI2* is a gene which is induced by EBV infection and which has been found to be upregulated in a variety of autoimmune diseases. EBI2 is a critical gene in immunity and central nervous system function; it is a negative regulator of the innate immune response in monocytes. Its heterogeneous expression in CFS/ME may indicate an ongoing host response to EBV reactivation and on this basis, could explain the heterogeneous occurrence of many of the immune and neurological abnormalities reported in CFS/ME patients. The EBI subtype may account for 38–55% CFS/ME patients. *EBI2* antagonists may hold promise for the treatment of CFS/ME patients of the EBI subtype. Further work is required to confirm the role of EBV and of *EBI2* and its oxysterol ligands in CFS/ME, and to identify the most practical means to identify patients of the EBI subtype.

## Data Availability

All relevant data is contained within the manuscript.

## Author Contributions

JK conceived the idea for this review, collated the data, and wrote the paper, without assistance from any other person.

### Conflict of Interest Statement

The author declares that the research was conducted in the absence of any commercial or financial relationships that could be construed as a potential conflict of interest.
